# Urinary Incontinence in Women: Modern Methods of Physiotherapy as a Support for Surgical Treatment or Independent Therapy

**DOI:** 10.3390/jcm9041211

**Published:** 2020-04-23

**Authors:** Agnieszka Irena Mazur-Bialy, Daria Kołomańska-Bogucka, Caroline Nowakowski, Sabina Tim

**Affiliations:** Department of Biomechanics and Kinesiology, Faculty of Health Science, Jagiellonian University Medical College, Grzegorzecka 20, 31-531 Krakow, Poland

**Keywords:** incontinence, physiotherapy, biofeedback, electrostimulation, vibration, magnetic stimulation, pelvic floor muscle training

## Abstract

Urinary incontinence (UI) is a common health problem affecting quality of life of nearly 420 million people, both women and men. Pelvic floor muscle (PFM) training and other physiotherapy techniques play an important role in non-surgical UI treatment, but their therapeutic effectiveness is limited to slight or moderate severity of UI. Higher UI severity requires surgical procedures with pre- and post-operative physiotherapy. Given that nearly 30%–40% of women without dysfunction and about 70% with pelvic floor dysfunction are unable to perform a correct PFM contraction, therefore, it is particularly important to implement physiotherapeutic techniques aimed at early activation of PFM. Presently, UI physiotherapy focuses primarily on PFM therapy and its proper cooperation with synergistic muscles, the respiratory diaphragm, and correction of improper everyday habits for better pelvic organ support and continence. The purpose of this work is a systematic review showing the possibilities of using physiotherapeutic techniques in the treatment of UI in women with attention to the techniques of PFM activation. Evidence of the effectiveness of well-known (e.g., PFM training, biofeedback, and electrostimulation) and less-known (e.g., magnetostimulation, vibration training) techniques will be presented here regarding the treatment of symptoms of urinary incontinence in women.

## 1. Introduction

Urinary incontinence (UI), characterized by involuntary loss of urine (ICS—International Continence Society), is a serious social and health issue whose incidence is increasing [1]. UI is diagnosed more often in women than in men [2], and in 2018, it was predicted to affect nearly 420 million people (300 million women and 120 million men) worldwide [1]. However, this number may be underestimated because of the intimate nature of UI.

The main types of UI are stress UI (SUI), urge UI (UUI), and mixed UI (MUI) [3]. Briefly, SUI is characterized by loss of urine as a consequence of an increase in intra-abdominal pressure, which results in an increase in intravesical pressure that exceeds the maximum urethral closure pressure. This situation occurs, for example, during coughing, sneezing, or jumping [4]. In UUI, loss of urine occurs with sudden, strong urgency that is inadequate to the degree of bladder filling [5]. MUI combines the symptoms of the above two types, and the involuntary loss of urine is associated with urgency and/or increase in intra-abdominal pressure [6].

The etiology of UI is multifactorial, and its causes are mainly associated with the dysfunction of the bladder, pelvic floor muscles (PFMs) [7], the ligament apparatus [8], connective tissue including, inter alia, endopelvic fascia and/or neural structures [9]. The risk of UI increases with age [10,11], but it can also occur after delivery as well as in young nulliparous women [12]. The main risk factors of UI development include the following: predisposing factors, such as genetic factors and gender, factors associated with damage of the continence mechanism (e.g., abdominal surgery, multiple births), and promotional factors, such as overweight/obesity, menopause, drugs, and urinary tract infections [13]. UI treatment includes surgical and conservative methods, among which physiotherapy is recommended as the first line of therapy because of its effectiveness, low cost, and low risk associated with it.

## 2. Incontinence—Impact on Quality of Life and Economic Status

Studies show that UI significantly reduces women’s quality of life [14,15,16,17]. Shame, anxiety, and fear of unpleasant smell or uncontrolled loss of urine lead to withdrawal of the affected women from social life. Women suffering from UI are characterized by a higher degree of emotional disorders than those with normal micturition [18,19]. According to Felde et al. [20], women with UI are more likely to suffer from depressive disorders and anxiety. UI also has a negative effect on the women’s sex life [21,22] and reduces self-esteem [23]. Women with UI avoid sexual intercourse due to the possibility of urine leakage. Nearly 50%–68% of women with incontinence have been shown to suffer from sexual dysfunction [22]. With the development of UI, the risk of falls and injuries due to frequent necessity to use the toilet increases [24]. Older women diagnosed with UI are 1.5 to 2.3 times more likely to fall, which results in a worsening of overall health and increased healthcare costs [25]. In addition, because of fear of lack of control over voiding, the level of physical activity is also reduced [14]. Attention should also be paid to reduction in the economic level of patients and high treatment costs [16]. UI has a negative effect on women’s working lives (absenteeism, lower efficiency, and lower work pace) [17]; moreover, expenses for the purchase of underwear, absorbent pads, and diapers are increasing [26]. The average annual cost of treatment of women with UI in Germany was € 515, in Spain € 655, and in Great Britain/Ireland € 395, where absorbent pads accounted for nearly 51% of the treatment cost [27]. Koening et al. estimated that the total annual cost of treatment for one woman with UI is nearly 22% higher than the average cost of treatment for the general female population in 2017 in Switzerland [28].

## 3. Role of Pelvic Floor Muscle in Continence

The pelvic floor (PF) consists of passive (ligaments, fascia) and active (muscles) components that support the bladder, reproductive organs, and rectum [29]. The proper cooperation between the soft tissue components and the appropriate action determine the proper closing and opening of the key areas such as the urethra (micturition), vagina (delivery), or rectum (defecation). Therefore, in addition to maintaining organ statics, they also affect the proper course of labor, voids, or defecation as well as fecal and urine continence [30,31]. 

Studies have shown that pelvic floor muscles (PFMs) together with internal oblique, intercostal muscles, transverse abdominal muscle, and diaphragm are responsible for maintaining proper body posture and breathing [32,33]. Due to numerous myofascial connections between PFM and other muscles all movements are functionally linked, and throughout this system, PFM exists as the basis for local stabilization [33,34]. For this reason, myofascial disorders as well as posture disorders can lead to impaired PFM function and, consequently, their weakness and UI [35]. 

In physiotherapeutic examination PFM function is evaluated using the PERFECT scale assessing strength of voluntary contraction (P), endurance (E), slow-twitch muscle fiber performance (R), fast-twitch muscle fiber performance (F), PFM contraction pattern (E), co-contraction of the transverse abdominal muscle (C) and involuntary contraction in response to increased intra-abdominal pressure (T) [36,37]. PFM strength is assessed using a 6-stage Modified Oxford Scale [36,38]. The most common diagnoses are overactive or underactive PFM. An overactive PF is not able to relax after any contraction or in a situation where relaxation is required, e.g., voiding or defecation, while underactive PFM is unable to perform voluntary or involuntary contraction necessary for, e.g., pelvic organ stabilization against lowering during increasing of intra-abdominal pressure [3,39]. 

The improper PFM tonus leads to many disfunction [29,40]. Research indicates that increased tone of PFMs can prolong delivery course, while simultaneously favoring greater damage within them [41,42]. PF hypertonicity frequently occurs in women with chronic pelvic pain [40], but may also leads to incontinence caused by increased intra-abdominal pressure. It is known that the persistent state of excessive muscle tension leads to a weakening of their strength and endurance. Excessive tension of the pubic-rectal muscle, by reducing the anorectal angle, may also result in hindered defecation and increases the need for displacement using a stronger abdominal compressor [43]. Similarly, in the case of voiding, excessive PFM tension may functionally hinder its initiation, resulting in the necessity of using the abdominal pressure [44]. This condition also favors the occurrence of constipation and, consequently, may increase the risk of hemorrhoids [44]. Abuse of the abdominal press is also one of the factors increasing the risk of lowering within the pelvic organs [45]. Therefore, control and normalization of PFM tone can be an important factor for maintaining the functional balance of the complex [46].

## 4. Materials and Methods

The aim of this paper is a systematic review showing the possibilities of using physiotherapeutic techniques in the treatment of UI in women with attention to the techniques of PFM activation. The review of the literature was carried out in the Medline-PubMed database. Keywords varied depending on the therapy: PF training AND urinary incontinence; electrotherapy AND urinary incontinence; urinary incontinence AND magnetic stimulation OR Extracorporeal Magnetic Innervation; whole-body vibration OR vibrance OR vibration OR perineal stimulation OR intravaginal vibratory AND urinary incontinence. 

Inclusion and exclusion criteria. The review included works that were published in the years 2009–2020 in English. The research should have been carried out on women with diagnosis of UI. Publications should investigate the impact of various physiotherapeutic methods on the treatment of UI. Exclusion criteria included studies not related to women with UI, publications in English made or published before 2009, non-RCTs studies and study protocols. Other exclusion criteria were pregnancy or puerperium period < 6 weeks, cancer, neurological and spine diseases, no access to the full-text version of articles.

## 5. Results

**Figure 1:**
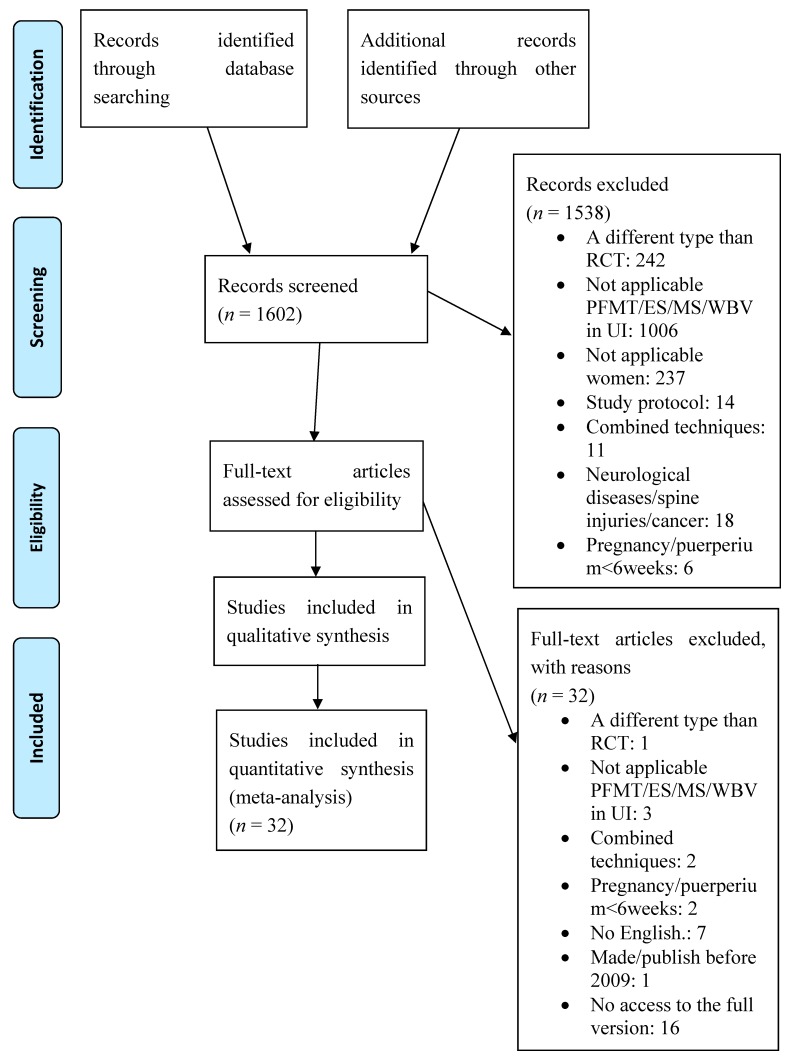
A diagram showing the stages of the literature review (2009 PRISMA flow diagram).

A total of 1602 references were found. Based on the analysis of titles and abstracts, 1538 publications were rejected. A total of 64 articles were left to read in full. Finally, 32 papers were qualified for the review (Figure 1): 11 publications on PFM training, 8 on electrical stimulation, 6 on magnetic stimulation, and 7 on whole-body vibration training were qualified for review.

## 6. Physiotherapeutic Techniques in UI

### 6.1. Pelvic Floor Muscle Training 

Pelvic floor muscle training (PFMT) is a well-described, effective, and most commonly used method of physiotherapeutic UI treatment which has been recognized and recommended as the first line of conservative SUI treatment [47]. For the first time, PFM exercises were described in 1948 by Arnold Kegel as a behavioral method in the treatment of UI [48]. Their effectiveness is proven regardless of age and BMI [49]. PFMT is based on two pelvic muscle functions: pelvic support and cooperation in the urethral sphincter closing mechanism [50]. A PFMT can be performed to increase strength, endurance, and muscle coordination [51]. The studies estimate that regular PFMT deceased loss of urine and improved quality of life [52]. However, research shows that nearly 30%–40% of women are unable to perform the correct voluntary PFM contraction despite the instruction [53] and in the population of women with pelvic floor dysfunction, this value increases to 70% [54]. In this situation, it is necessary to use facilitating techniques for the teaching of PFM voluntary contraction, because PFMT alone will not yield the assumed outcomes [46]. 

De Andrade et al. confirmed that giving only the instruction of PFM exercise increased knowledge about the PF but was not effective in contracting and strengthening the PFM [55]. However, there are also studies that confirm the effectiveness of PFMT performed at home with app-based audio guidance after prior instruction. Exercises with voice guidance are more effective than conventional exercises [56].

Unfortunately, most randomized studies on the effectiveness of PFMT include patients who can perform voluntary PFM contraction in their inclusion criteria [57,58]. According to Mateus-Vasconcelos et al. [59], effective facilitating techniques include vaginal palpation with or without posterior pelvic tilt and electrostimulation, however, with much lower efficiency. These techniques should be used when the strength of PFM contraction is 0–1 on the Modified Oxford Scale (MOS) [59]; patients with higher MOS value (2 and more) respond well to PFMT therapy [57,58]. It should also be noted here that PFMT can be conducted both in a form supervised by a physiotherapist as well as in an unattended form at the patient’s home. Studies have shown that unattended training is significantly less effective at relieving UI symptoms than supervised training [60]. However, the effectiveness of home unsupervised training can be increased using additional techniques such as electrostimulation [61], biofeedback (BF) [62], or vaginal cones [63]. A detailed description of the studies included in the review is presented in Table 1.

Studies show that the effectiveness of PFMT increases significantly if the BF technique is used. It should be emphasized that BF is not a method of therapy but a form of its support, allowing for a better feeling and imaging of the structures and muscles that we want to activate and strengthen. Currently, in PFMT, it is possible to use many forms of BF; the most common is the surface electromyography (sEMG) method using a vaginal probe that allows the physiotherapist to read the electrical activity of the PFMs [69] and use this information to design exercise tasks, programs, or games for the patient depending on her level of advancement. Because of the use of BF, patients can correctly identify contracting muscles and perform their activity depending on the training task, which is usually illustrated by animation or playing a game on the screen. Other forms of BF currently used in urogynecological physiotherapy is pressure BF using a vaginal probe that reads pressure changes caused by PFM contraction—the principle of its operation is analogical to the perineometer [70]. PFM contraction can also be visualized using BF combined with ultrasound, which is so-called “sonofeedback” [71]. The effect of exercise with biofeedback on UI has been the subject of numerous studies [63,72], which showed that BF can be an effective support for the training process. 

The other type of training PFM can be hypopressive exercises (HE) proposed by Caufriez. HE relies on the reflex activation of PFM through adequate breathing and body position changes. They also activate the transverse abdominal muscle, increase PFM endurance, but do not lead to their hypertrophy [73]. An important factor affecting the effectiveness of PFM training is the proper positioning of the pelvis and ankle. Studies show that a greater contraction of PFM and postural muscles occurs during ankle dorsiflexion [74]. These studies provide a valuable tip for conducting the most effective PFM exercises. A detailed description of the studies included in the review is presented in Table 2.

### 6.2. Manual Therapy

Pelvic Floor Dysfunction (PFD) e.g., urinary incontinence is difficult to treat because of the many entangled factors: urologic, gynecologic, psychologic, and musculoskeletal factors [79]. The PF, as with any skeletal muscle, may have increased or decreased muscle tone [80]. Hypo- and hypertonia of PF causes various ailments that should be treated individually [81]. It should be also remembered that organs and muscles are connected by fascia. Endopelvic fascia is continuous with visceral and abdominal fascia, diaphragm, posterior intermuscular septum, and fascia of adductors. Any disorders in hip, core muscles may affect PFD [80]. 

There are studies confirming effectiveness of dry needling [80], trigger points (TrP) releasing [82,83] and massage [84] in reducing urinary incontinence symptoms and pelvic pain. TrP manual compression and dry needling result in softening of the taut band, oxygenate the problem muscles area, relieve pain, and improve disturbed movement patterns [83]. Massage is also recommended in PFD treatment. Studies indicated that massage of the abdominal muscles and directly of urinary bladder improves bladder function and blood distribution in this area. Then occurs cell regeneration, muscle elasticity and contractility increase, and muscle tone normalizes [84].

### 6.3. Electrical Stimulation

Electrical stimulation is one of the most commonly used therapeutic methods in the treatment of UI [85]. The method of electronic PFM stimulation was first described in 1963 by Caldwell. It is a noninvasive, passive treatment that induces muscle contraction [86]. Electrical stimulation can be an individual therapy or can be combined with PFMT or BF, which, according to research, significantly increases its effectiveness not only in urine but also in fecal incontinence [87]. However, because of pain or discomfort experienced by the patient during the procedure, it is not recommended as the first line of UI treatment [88]. The arrangement of the electrodes depends on the type of target tissue. For muscle tissue, the electrodes should be located on the muscle belly, while in the nerve tissue, they should be located along the course of the nerve or one electrode at the motor’s nerve point and the other on the target muscle [89]. Current flow causes contraction of the PFMs with simultaneous inhibition of detrusor muscle activity [90]. Distinguished was transvaginal [91] as well as surface electrical stimulation [61].

Electrical stimulation does not stimulate the muscle directly, but through motor nerves. Therefore, frequencies above 70 Hz may cause neuromuscular damage [92]. The optimal stimulation frequency is 50 Hz for SUI [88] and 10–20 Hz for UUI [93]. The selection of appropriate parameters in electrotherapy is required to obtain an increase in muscle strength [94]. Electrical stimulation significantly reduces the symptoms of UI [61]. A significant reduction in UI symptoms was noted after both surface electrostimulation and transvaginal electrical stimulation [95,96]. Pereira et al. [96] noted a significant reduction of urinary loss events after 6 weeks of electrotherapy in women with SUI compared to that in patients without treatment. In addition, electrical stimulation is characterized by a low risk of side effects. In the study by Alves et al. [97], no side effects, such as pain, discomfort, or vaginal infection were noted in patients in both groups. Franzén et al. [98] examined the long-term effect of electrostimulation on the occurrence of UI. After 6 months from the end of therapy, nearly 73% of the examined patients reported an improvement in continence [98]. The long-term effect of electrostimulation has also been studied by Fürst et al. [99]. After 3 months, a significant increase in the time between voids was observed in the examined patients [from 2.24 ± 1.09 to 3.35 ± 0.86 h (*p* < 0.0001)]; similar differences were, however, not observed after 96 months [99]. A detailed description of the studies included in the review is presented in Table 3.

### 6.4. Magnetic Stimulation

The use of magnetic stimulation (MS) or extracorporeal magnetic innervation (ExMI) in non-surgical treatment of UI has raised extensive controversy, but recent studies have provided evidence of the effectiveness of this therapy. In 1998, PFM magnetic stimulation was approved by the US Food and Drug Administration (FDA) as a conservative treatment method of UI [102] and was indicated among non-surgical UI therapies in the latest guidelines of European Association of Urology [103]. Although the European Association of Urology (EAU) 2018 guidelines, based on evidence from 2007 and 2008 (two papers), do not recommend the use of MS in adult women with SUI (level of evidence: 2a), it however omits an important study conducted recently, which will be presented below. MS is a noninvasive, passive method of stimulating the roots of the sacral nerves or PFMs [104]. The MS method uses a special chair with a therapeutic head placed in the seat; as the magnetic field can penetrate through clothes, stimulation can be carried out in clothing, which significantly increases the comfort of therapy [105]. The head creates a magnetic field that penetrates the pelvic organs, acting directly on the motor fibers of the nerves. MS stimulates the PFMs to repeatedly contract and relax, which are clearly experienced during the procedure [106]. Moreover, MS-induced PFM contraction simultaneously inhibits the reflex mechanism of emptying the bladder [107], and consequently leads to an increase in bladder capacity in both female bladder outlet obstruction [108] as well as in UUI or SUI [109]. MS also increases the strength of PFMs [110], with no adverse effects [103,111]; level of evidence: 1b. In addition, MS therapy significant decreases the intensity of urinary incontinence symptoms in women with SUI [112,113] and UUI [109] measured in 1 h pad test or voiding diary. The beneficial effect of MS was also noted in the reduction of UI incidents in patients after radical hysterectomy due to cervical cancer [114]. Moreover, it also causes short- and long-term improvement in the quality of life of women with SUI [115] while improving the severity of depression symptoms observed in these women [106,116]. It is also important to highlight the findings of Koh et al. [108] who showed comparable efficacy of Extracorporeal Magnetic Innervation (ExMI) as alpha-blocker monotherapy in the treatment of female bladder outlet obstruction. According to Weber-Rajek et al. [106], ExMI leads to a significant decrease in myostatin levels in patients with SUI; myostatin is an inhibitor of myogenesis [117,118], which may play an important role in the remodeling and regeneration of PFM. For an optimal therapeutic effect, the recommended dose of magnetic field is between 5 and 50 Hz [112,113,119] and it is dependent on the intended purpose of the therapy. Lower frequencies (5–10 HZ), as in ES, are used to inhibit detrusor activity, while higher (20–50 Hz) are effective in stimulating PFM contraction and closing the urethra [112,120]. MS is an alternative to electrical stimulation (ES); however, compared to ES, it is a painless method and more comfortable for the patient [105]. MS is assessed by patients with SUI as a well-tolerated and satisfactory method [121]. A detailed description is presented in Table 4.

### 6.5. Whole-Body Vibration

Whole-body vibration training (WBV) is a valuable tool supporting both sports training and physiotherapy [124]. The use of a vibrating platform in exercises supports the therapeutic process in patients with nonspecific lumbar pain [125], multiple sclerosis [126], Parkinson’s disease [127], or hemiplegia [128]. The method consists of carrying out a set of exercises performed on a vibrating platform that oscillates at a given frequency and amplitude [129]. Mechanical vibration conducted during exercise leads to a change in muscle length. Information about muscle lengthening is transmitted by sensory nerves to the spinal cord and consequently provokes muscle contraction through the activity of α-motor neurons [130]. The induction of myoelectric activity during WBV is well documented [131,132]. Moreover, studies have confirmed that high-intensity synchronous WBV (frequency 40 Hz, amplitude 4 mm) with a long duration (60 and 90 s) elicits a response from PFM, leading to an increase in the mean amplitude of the sEMG signal from PFMs in young continent women [133]. Moreover, studies have indicated that the activation of PFM is caused by both sinusoidal (S-WBV) and stochastic resonance WBV (SR-WBV), and the level of PFM activation depends on the intensity of the vibration [134]. It has also been shown that SR-WBV-induced PFM contraction is higher than voluntary muscle contraction, which is particularly pronounced in the case of PFM weakened by delivery [134]. However, there were no differences in efficiency between continuous and intermittent RS-WBV [130]. WBV also exerts positive effects on dysfunctional PF in women with UI. Farzinmehr et al. [135] showed that 4-week WBV training has a comparable therapeutic effect to PFMT, causing a significant reduction in UI symptoms and improving the quality of life of patients, which was also maintained in the 3-month follow-up. A detailed description of the studies included in the review is provided in Table 5.

A slightly different form of vibration therapy is the use of techniques directly affecting PFM using a transcutaneous vibratory perineal stimulation [136] or intravaginal vibratory stimulation [137,138,139]. These techniques use different stimulation parameters from those used in the indirect techniques described above for review see [129,140]. Nevertheless, studies show that the intravaginal vibratory stimulation significantly increase the effectiveness of PFMT [137], while being more effective in improving PFM strength than transvaginal electrostimulation when applied in women with SUI. Hence, it can be considered to be a support for the therapeutic process in patients with PFM or UI dysfunction. An overview of the mentioned articles is presented in Table 6 below.

## 7. Conclusions

As indicated above, physiotherapy is certainly an important element of conservative treatment of UI; however, its effectiveness is limited to less advanced conditions. In advanced UI stages, however, physiotherapy is an important element in preparing the patient for surgical treatment, significantly increasing the success of surgery. Moreover, physiotherapy significantly improves the quality of life of women with UI [136]; however, to achieve optimal therapeutic effects, it is sometimes beneficial to use a combination of several physiotherapeutic techniques. PFMT and ES are the most commonly used therapies for treating UI. In the first line EAU recommends PFMT to prevent and treat UI (level of evidence: 1). However, for many women, isolated voluntary PFM contraction is a major concern. Studies show that often patients with UI are unable to perform isolated voluntary and involuntary PFM contraction; hence, in this case, basing the therapy only on exercises will be ineffective. In these situations, it becomes extremely important to use techniques focused on PFM sensitization. Sometimes it will be necessary to use palpation or apparatus techniques, but without PFM activation, the implementation of training programs will be ineffective. Electrical stimulation [59,95,96,97,98,99,100,101] and biofeedback [57,75,78] can be used in addition to PFMT or to help women to initiate a contraction and improve therapeutic effects (level of evidence: 2). In such cases, it is a preferable activation of PFM to fully use the potential of the female pelvic floor. Recent studies [112,116,122] suggest that MS induces improvement in the severity of incontinence (level of evidence: 2); however, EAU does not recommend using magnetic stimulation to treat UI (strong evidence) and they do not mention vibration training [103]. EAU guidelines do not include the latest RCT work indicating the effectiveness of the above techniques in reduction of UI symptoms, these works are presented in this review. Our results are familiar with other reviews regarding physiotherapeutic methods in UI (Table 7). Given the above, it can be concluded that physiotherapy is an important and effective element of therapy in patients with UI, with high levels of evidence. Nevertheless, the still small number of RCTs makes further research necessary to increase their reliability and improve the effectiveness of therapy.

## Figures and Tables

**Table 1:** Characterization of selected studies on the effects of pelvic floor muscle training on the pelvic floor muscles (PFM) activity and/or severity of urinary incontinence symptoms.

Reference	Main Objective	Patients Characteristic	Study Description	Outcome
Alves et al. (2015) [58]	Verification of the effect of PFMT to increased PFM contraction and decreased anterior POP in postmenopausal women.	30 postmenopausal women Intervention: 18 (aged 66.11 ± 8.72 years) Control: 12 (aged 65.67 ± 9.21 years)	Control: 12 fitness session for 60 min, twice a week, per 6 weeks, instruction about the PFM function and the correct way to contract it, without PFMT. Intervention: Individually teaching the exercises to follow with the Gym Ball, pelvic mobility, stretching, strengthening and relaxation exercises in five positions (supine, sitting on the floor, on the Gym Ball, squat and standing), with the PFM contractions (8 s hold—16 s relax). 12 sessions of 30 min, twice a week, six weeks treatment. Assessment: sEMG, MOS, ICIQ UI-SF, ICIQ-OAB, ICIQ-VS	PFMT increased PFM contractility and decreased anterior pelvic organ prolapse and urinary symptoms.
McLean et al. (2013) [64]	Verification of the effect of PFMT on urethral morphology and mobility in women with SUI.	35 women with SUI Control: 17 (aged 54.0 ± 8.4 years) Intervention: 18 (aged 49.5 ± 8.2 years)	Control: no intervention Intervention: supervised PFMT 30 min per day, for 12 weeks Assessment: MOS, 3-day bladder diary, pad test, UDI-6, IIQ-7, ultrasound evaluation	The intervention group showed a reduced bladder neck mobility during coughing and increased cross-sectional area of their urethra (from baseline to the end of the study). Similar results were not obtained in the control group. Intervention group showed statistically significant changes in urine leakage (3-day bladder diary) and in results of IIQ-7. Similar significance was not obtained for pad test and UDI-6. The control group did not achieve significant improvement in any parameters.
Celiker Tosun et al. (2015) [65]	Checking whether pelvic floor muscle training and increasing PFM strength will eliminate UI symptoms.	121 women Intervention group (IG): 58 (aged 51.7 ± 10.3) Control group (CG): 63 (aged 52.5 ± 9.1)	Intervention group: For the first two weeks, patients had 3 sessions (30 min) with a physiotherapist. They received a posture training. Then individual PFM exercise schedules were designed for patients (home-based exercise program). This part of study lasts 12 weeks. If someone did not increase muscle strength to 5 on the Oxford Scale, they could continue participating in the study. Control group: no treatment Assessment: IIQ-7, UDI-6, bladder diary, stop test, pad test, the PERFECT scale, the perineometer, and ultrasound, the Oxford Scale	The IG group demonstrated a significant improvement in all examined aspects after study. Similar results were not obtained in CG group. In the intergroup analysis comparing the results after 12 weeks of the study, the training group achieved a significant improvement in the parameters tested compared to the control group. All people who continued to exercise to achieve PFM strength on 5 on the Oxford Scale obtained a significant improvement in UI symptoms.
Nascimento-Correia et al. (2012) [66]	Assessment of effects of kinesiotherapy on function and level of pressure of PFM and quality of life of women with UI.	30 women Intervention group (IG): 15 (age 60.20 ± 8.16) Control group (CG): 15 (age 61.53 ± 10.12)	Intervention group: 12 sessions of one-hour PFMT, once a week Control group: no treatment Assessment: the 1 h pad test, KHQ, the PERFECT scale, the perineometer	The IG group showed a significant improvement in urinary leakage (pad test), function of PFM (the PERFECT scale) and PFM pressure (the perineometer) and in some aspects of KHQ. Similar results were not obtained in CG group. In the intergroup analysis in all the above aspects statistically significant differences were obtained in favor of the IG group than CG.
Pereira et al. (2011) [67]	Assessment of effectiveness of PFMT in group treatment sessions (GT), individual sessions (IT) and control group (CG) in women with SUI.	45 women GT group: 15 women (age 60.2 ± 8.16) IT group: 15 women (age 60.6 ± 12.63) Control group: 15 women (age 61.53 ± 10.11)	Intervention: GT group—PFMT group treatment session, IT group—PFMT individual treatment session, Control group—no treatment. Exercises in the training groups were performed for 6 weeks, 2 sessions a week for 1 h. Assessment: KHQ, a perineometer, the PERFECT scale, MOS, the 1 h pad test	IT group obtained a significant decrease in urinary loss (pad test). A similar effect was obtained in the intergroup analysis (GT vs. CG, IT vs. CG). GT and IT group presented a significant increase of the pressure perineometry of PFM. After the treatment, significant differences in this aspect were demonstrated between the groups GT vs. CG and IT vs. CG. Muscle strength increased significantly after therapy only in group GT and IT (the 6-point Modified Oxford Scale).
Kashanian et al. (2011) [68]	Assessment of the effectiveness of PFM training without or using a resistance device in women with UI.	85 women with UIAPFMT (assisted PFMT) group: 39 women (age 39.07 ± 6.18) PFMT group: 46 women (age 40.56 ± 6.18)	PFMT group: Kegel exercises—contraction: relaxation 6–8 s: 6 s. Time: 15 min, 2 times a day for 12 weeks. APFMT group: after PFMT, the Kegelmaster was used twice a day for 15 min of each session for a total of 12 weeks. Assessment: MOS, I-QOL, IIQ, UDI, VAS	In both groups there was an improvement in UI severity, number of UI episodes, PFM strength and participation in social life (these were not significantly different in intergroup analysis). Compared to the PFMT group, side effects occurred in the APFMT group (vaginal discharge, pain, spotting).

UI—urinary incontinence, PFMT—Pelvic Floor Muscle Training, sEMG—surface electromyography, MOS-Modified Oxford Scale, ICIQ UI-SF—International Consultation on Incontinence Questionnaire—Short Form, ICIQ-OAB—International Consultation on Incontinence Questionnaire Overactive Bladder, ICIQ-VS—International Consultation on Incontinence Questionnaire on Vaginal Symptoms, IIQ-7—Incontinence Impact Questionnaire-7, UDI-6—Urogenital Distress Inventory-6, I-QOL—Incontinence Quality of Life, VAS—Visual Analog Scale.

**Table 2:** Characteristic of selected studies on the effects of pelvic floor muscle training and biofeedback on the pelvic floor muscles (PFM) activity and/or severity of urinary incontinence symptoms.

Reference	Main Objective	Patients Characteristic	Study Description	Outcome
Bertotto et al. (2017) [75]	Evaluation of the effectiveness of PFM training with or without biofeedback in improving muscle strength, myoelectric activity, pre-contraction, and quality of life in postmenopausal women with SUI.	45 women Control group: 14 (age 57.1 ± 5.3) PFMT group: 15 (age 59.3 ± 4.9) PFMT + BF group: 16 (age 58.4 ± 6.8)	Intervention: PFMT group—PFM exercises—8 sessions, twice a week for 20 min for 4 weeks, PFMT + BF group—8 sessions of PFM exercises with BF, twice a week for 20 min for 4 weeks, Control group—no treatment. Assessment: ICIQ-SF, MOS, electromyography.	The PFMT and PFMT+BF group present a significant improvement in muscle strength as compared to control group (the Modified Oxford Scale). Based on EMG, better PFM function was achieved in groups PFMT and PFMT+BF. The PFMT+BF group also achieved significant improvements compared to the PFMT group (the Modified Oxford Scale, EMG). Significant improvement was also obtained in QOl (only in PFMT and PFMT+BF groups).
Fitz et al. (2017) [76]	Assessing whether biofeedback added to pelvic floor muscle training increases exercise frequency in women with SUI.	72 women with SUI PFMT group: 37 (age 56.6 ± 12.0) PFMT + BF group: 35 (age 56.1 ± 10.5)	Intervention: Both groups performed outpatient and home PFM exercises for 3 months, BF group also get a vaginal BF equipment. The outpatient trainings were supervised by a physiotherapist (24 session for 3 months, twice a week for 40 min). Home PFM exercises consisted of three sets of 10 repetitions daily for 3 months. From 4th to 9th months women from both groups performed PFM exercises only at home. Assessment: A voiding diary, a modified 20-min pad test, an exercise diary, the Oxford Grading Scale, a Peritron manometer, I-QOl.	Both groups present a significant reduction in the number of episodes of urine loss (a voiding diary), in the urine leakage (a pad test) (from baseline to 3 and 9 months). Both groups significantly improved a PFM function (PFMT group did not obtain a significant difference for the measurement from baseline to 9 months). Both groups showed significantly better quality of life (I-QOl). There were no significant differences between groups. There were no differences between groups in the number of monthly training, weekly frequency (days/week) and exercises sets per day after 3 months of supervised treatment and at 9-month follow-up.
Hirakawa et al. (2013) [77]	Comparison of the effectiveness of pelvic floor muscles training (PFMT) with or without biofeedback (BF) in women with SUI.	46 women PFMT group: 23 (age 58.3 ± 11.2) PFMT + BF: 23 (age 55.3 ± 9.8)	Intervention: PFM training (for 12 weeks) twice a day: 10 maximum contractions for 5 s + 10 s of relaxation. Then 10 quick maximum contractions with a 2 s hold + 4 s relaxation—performed twice with 1 min break between sets. Women from BF group also get EMG-assisted home training device. Assessment: KHQ, ICIQ-SF, a voiding diary, 1 h pad test, perineometer for PFM strength.	In both groups, there was a significant improvement in the women’s quality of life (KHQ) and subjective symptoms (ICIQ-SF). Reduction of UI episodes (voiding diary) were significant only in PFMT group, in BF group showed a tendency to decrease episodes of UI, but it was not statistically significant. Both groups showed a tendency to decrease the leakage volume (1 h pad test). In both groups a maximum vaginal pressure (perineometer) increased significantly. There were no significant differences between groups.
Huebner et al. (2011) [57]	Comparison of three different strategies: (1) EMG biofeedback-assisted PFMT and conventional ES; (2) EMG biofeedback-assisted PFMT and dynamic ES; and (3) EMG biofeedback-assisted PFMT for treatment of SUI.	108 women suffering from SUI	Group 1: EMG biofeedback-assisted PFMT and conventional ES (50 Hz, 20–80 mA, stimulation 8 s, rest 8 s, active contraction 8 s, rest 15 s), 15 min twice a day per 3 months Group 2: EMG biofeedback-assisted PFMT and dynamic ES (50 Hz, 20–80 mA, active contraction 8 s, after gaining the max contraction, ES was added, Stimulation 8 s, rest 15 s), 15 min twice a day per 3 months Group 3: EMG biofeedback-assisted PFMT (active contraction 8 s, rest 15 s), 15 min twice a day per 3 months Assessment: KHQ, number of pads, pad weight test, digital vaginal palpation, MOS, intravaginal EMG.	In all groups QOL (KHQ) significantly improved over the 12-week. In all three groups, the contractility of PFM (MOS, EMG) significantly increased. The number of pads used per day decreased and the pad weight test showed a significant improvement for every group. There are no differences between groups. The additional ES did not show any benefits.
Chmielewska et al. (2019) [78]	Comparison of the effectiveness of pelvic floor muscles training with surface electromyographic (sEMG) biofeedback (BF) with Pilates exercises (PG) in women with SUI. The second aim is to compare changes in parameters obtained in voids diaries and the quality-of-life questionnaire.	31 women with SUI BF group: 18 (age 52.9 ± 4) PG group: 13 (51.5–6 ± 5.2)	In both groups 24 sessions for 8 weeks (3 times a week). Before the training 3 instructional sessions (the same for both study groups) BF: time of session: 30–50 min. PFM strength training began with 80% of the MVC (3 s of contraction/6 s of relaxation). The number of short contractions increased accordingly in the following weeks (from 21 units to 60 units). Endurance training included 45 to 120 units (contraction/relaxation at 60% MVC with 90 s interval between series—contraction/relaxation for 5 s at 1–4 week and 10 s at 5–8 week). PG: time of one session: 40–50 min. The training consisted of Pilates exercises and voluntary PFM contractions. Assessment: KHQ, a voiding diary, sEMG, ICIQ-SF.	In both groups the number of episodes of urinary incontinence decreased at each measurement point. There were no differences between the groups in the frequency of urination and the number of episodes of incontinence. After 8 weeks of exercise and 6 months of observation, it was shown that the Pilates method better improves the quality of life of women with SUI than BF training (KHQ). In turn, ICIQ-SF showed similar effectiveness of both Pilates and BF trainings.

SUI—Stress urinary incontinence; PFMT—pelvic floor muscles training; BF—biofeedback; KHQ—the King’s Health Questionnaire, ICIQ-SF—International Consultation on Incontinence Questionnaire—Short Form, PFM—Pelvic muscles floor, I-QOl—the Incontinence Quality-of-Life Questionnaire, EMG—electromyography, ES—electrical stimulation, QOL—quality of life, MOS—Modified Oxford Scale

**Table 3:** Characteristic of selected studies on the effects of electrical stimulation on the pelvic floor muscles (PFMs) activity and/or severity of urinary incontinence (UI) symptoms.

Reference	Main Objective	Patient Characteristics	Study Description	Outcome
Alves et al. (2011) [97]	Comparison of the effectiveness of low or medium frequency intravaginal neuromuscular electrostimulation (NMES) in the treatment of SUI in women.	20 women with SUI, aged 42–64(G1) NMES+MF: 10 (G2) NMES+LF: 10	Time: 20 min, 2 times a week, for 6 weeks, NMES at max tolerable intensity: G1: biphasic frequency (2000 Hz), 100 ms, time on: off 4: 8 s modulation frequency: 50 Hz, G2: biphasic, 50 Hz, 700 ms, time on: off 4: 8 s Assessment: perineometry examination for PFM, 1 hpad test, VAS for UI discomfort, the voiding diary.	Both low and medium frequency stimulation significantly reduced the micturition frequency (voiding dairy), the intensity of urine loss (pad test), and the degree of discomfort associated with UI (VAS) as well as PFM straight improved (perineometer) as observed by comparing the results of pre- and post-treatment. There were no significant differences between groups, indicating comparable efficacy of both stimulations.
Correira et al. (2014) [95]	Evaluation of the effectiveness of surface versus intravaginal electrical stimulation (SES vs. IVES) in the treatment of SUI in women.	45 women with SUI Aged over 50SES = 15 IVES = 15 Control = 15	Time: 20 min, 2 times a week, for 12 weeks, ES at max tolerable intensity: Frequency: 50 Hz, Pulse time: 700 ms, Work/rest cycle: 4/8 s, rise/fall: 2/2 s SES: 4 surface electrodes: 2 in the suprapubic region and 2 medial to ischial tuberosity IVES: intravaginal electrode Control: no treatment Assessment: 1 h pad test, KHQ, PERFECT scheme, Modified Oxford Scale, and perineometer for PFM evaluation	Both SES and IVES significantly reduced the intensity of urine loss (pad test), and improved the patients’ quality of life (KHQ); however, only IVES significantly improved the strength of PFM (Oxford Scale). No differences were noted in the control group as well as between the IVES and SES groups except for the strength of PFM. Both IVES and SES improved urinary retention, but only IVES significantly improved PFM strength.
Dmochowski et al. (2019) [100]	Comparison of therapeutic efficacy and safety of self-administered external and intravaginal electrical stimulation (NMES) in women with SUI.	148 women with SUI, aged 18–65 that did not benefit from pretrial Kegel exercise SES = 74 IVES = 74	12 weeks, at home after professional instruction, once daily for 5 days per week SES: 30 min, 50 Hz, 620 µs, 0.5 s ramp up/down, on/off 5/5 s (INNOVO device) IVES: 20 min, 50 Hz, 300 µs, 1 s ramp up/down, on/off 5/10 s (iTouch device) Assessment: pad test, 7-day voiding dairy, I-QOL, PISQ-IR, PGI-I, adverse events assesses	In both groups, there was a significant reduction in both the frequency (voiding dairy) and severity of urine loss (pad test) in the number of used pads and the I-QOL result in relation to the initial value (with no clinically relevant differences between the groups). Both devices were well-tolerated, but external stimulation showed better compliance. The main side effects included discomfort, infection, and pain.
Pereira et al. (2012) [96]	Assessment of the effectiveness of superficial electrical stimulation (SES) in older women with SUI compared to no treatment.	14 women with SUI Aged over 60SES = 7 Control = 7	Time: 20 min, 2 times a week, for 6 weeks, ES at max tolerable intensity: Frequency: 50 Hz, Pulse time: 700 ms, Work/rest cycle: 4/8 s, rise/fall: no data SES: 4 surface electrodes: 2 in the suprapubic region and 2 medial to ischial tuberosity Control: no treatment Assessment: 1 h pad test, KHQ, PFM pressure.	In the SES group, there was a significant improvement in the loss of urine (pad test) and improved quality of life (KHQ) compared to the initial value and the control group. There were no differences between the SES and control groups in terms of PFM pressure.
Mateus-Vasconcelos et al. (2018) [59]	Assessment of the effect of vaginal palpation, vaginal palpation with posterior pelvic tilt, and vaginal electrical stimulation in facilitating voluntary contraction of the PFM in women.	132 women with week PFM (0–1 in Modified Oxford Scale), Aged > 18:PG (vaginal palpation) = 33; PTG (vaginal palpation with posterior pelvic tilt) = 33; ESG (intravaginal electrical stimulation) = 33; CG (control group) = 33	For PG, PGT, and ESG: 1 session per week (3 interventions) with no PFMT at home PG: bidigital vaginal palpation to proprioceptive stimulation: therapist request 3 series of 10 contraction (6 s) with 6 s rest, and 6 quick contraction on the end PTG: as above but with posterior pelvic tilt movement during PFM contraction ESG: 20 min, 50 Hz, 200 ms, 5:10 on: off CG: verbal instruction for daily PFM contraction Assessment: The Modified Oxford Scale, ICIQ-UI-SF	All groups reported significant improved in PFM contraction capacity (Oxford Scale), but PTG and PG groups were significantly better than other groups. UI symptoms in terms of frequency, severity, and impact on quality of life (ICIQ-UI-SF) was also improved in all groups, but the PG results were significantly better than those of the other groups. The results suggest that vaginal palpation with or without posterior pelvic tilt was more effective in PFM facilitating than electric stimulation.
Franzén et al. (2010) [98]	Assessment of whether electrical stimulation may be more effective than pharmacotherapy in a patient with UUI.	61 women with UUI, Aged ≥ 60ES = 31 T (Tolterodine) = 30	SES: Time: 20 min, 10 therapies, 1–2 times a week, for 5–7 weeks, Frequency: 5–10 Hz at max tolerable intensity: T: tolterodine SR 4 mg; 1 time per day (if intolerable, reduced to 2 mg) for 6 months For both: evaluation at 6 weeks and after 6 months Assessment: KHQ, 48 h bladder diary, use of pads,	In both the SES and T groups, there was a significant decrease in the number of voids and a significant improvement in the quality of life of patients. There were no differences between the effectiveness in the ESE and T groups.
Fürst et al. (2014) [99]	Comparison of therapeutic efficacy of intravaginal electric stimulation (IVES) alone or IVES electrical stimulation combined with PFMT in women with SUI.	35 women with SUI, Aged 49.6 ± 10.6IVES + PFMT = 17 IVES = 18	IVES parameters: 30 min, 2 times a week for 3 months 4 Hz (15 min, 1 ms), 50 Hz (15 min, 700 µs), 20 mA, work/rest cycle: 4/8 s PFMT: 30 min, twice a week under the supervision of a physiotherapist on alternatives to IVES Assessment: Urodynamic examination, a voiding diary, PFM strength measured by maintenance of intravaginal cones.	In both groups, after 3 months, there was a significant increase in time between voids and a reduction in nocturia and urinary loss episodes. There were no differences between the groups. The results suggest that the combination of IVES with PFMT does not increase the effectiveness of SUI symptom treatment according to the analyzed parameters.
Schreiner et al. (2010) [101]	Evaluation of the efficacy of transcutaneous electrical stimulation of the tibial nerve (TTNS) for the treatment of UUI in older women.	51 women with UUI, aged TTNS: 26 Control: 26	In both groups, all patients were instructed to perform Kegel and bladder training for 12 weeks. In the TTNS group, patients were also subjected to electrical stimulation of the tibial nerve for 30 min, once a week for 12 weeks (10 Hz, 200 ms, 10–50 mA). Assessment: physical examination, 3-day voiding dairy, KHQ, ICIQ-UI-SF.	In both groups, there was a significant improvement in reducing the frequency of voiding episodes compared to pre-intervention values. However, the final values obtained in the electrostimulation group were significantly lower than those in the control group with Kegel and bladder training alone.

SUI—stress urinary incontinence; NMES—neuromuscular electrostimulation; PFM—pelvic floor muscle; UI—urinary incontinence; SES—surface electrical stimulation; IVES—intravaginal electrical stimulation; KHQ—King’s Health Questionnaire; I-QOL—Incontinence Quality-of-Life Scale; PISQ-IR—pelvic organ prolapse incontinence sexual questionnaire IUGA revised PGI-I Patient Global Impression of Improvement; ICIQ-UI-SF—International Consultation on Incontinence Questionnaire—Short Form; UUI—urge urinary incontinence; TTNS—transcutaneous electrical tibial nerve stimulation.

**Table 4:** Characteristic of selected studies on the effects of magnetic stimulation on the pelvic floor muscle (PFM) activity and/or severity of urinary incontinence (UI) symptoms.

Reference	Main Objective	Patients Characteristic	Study Description	Outcome
Weber-Rajek et al. (2018) [106]	Evaluation of the effectiveness of extracorporeal magnetic innervation (ExMI) in the treatment of women with SUI.	52 women with SUI, aged 61–76ExMI = 28 Control. = 24	ExMI: 15 min, 3 times a week, for 4 weeks, 2.0 T at 50 Hz, for 8 s on/4 s off, increasing from 20% to 100% in subsequent sessions Control: without intervention Assessment: QUID, GSES, RUIS, BDI, Myostatin level	In ExMI, but not in control group, the UI severity, depression, and myosin level were significantly reduced relative to the initial value. There were no differences in the final test values between the ExMI and control groups.
Weber-Rajek et al. (2020) [116]	Evaluation of the effectiveness of PFMT and ExMI in the treatment of women with SUI.	111 women with SUI, aged 45–77 PFMT: 40 ExMI: 37 Control: 34	PFMT: supervised 12 sessions, 45 min, 3 times a week for 4 weeks ExMI: 12 sessions, 15 min, 3 times a week for 4 weeks (2.0 T, 50 Hz, for 8 s on/4 s off, increasing from 20% to 100% during the subsequent sessions Control: without intervention Assessment: RUIS, BDI-II, GSES, KHQ	Both the PFMT and ExMI intervention induced significant improvement in the severity of UI (RUIS) and a reduction in depression symptoms (BDI-II), while improving the quality of life (KHQ). In addition, ExMI treatment improved self-efficacy beliefs (GSES) of patients with SUI.
Lim et al. (2017) [122]	Evaluation of the effectiveness of pulsed magnetic stimulation in the treatment of SUI as a non-surgical treatment method.	120 women with SUI, aged over 21 MS = 60 Sham = 60	MS: 16 or 32 sessions, 20 min, twice a week, 50 Hz, for 8 s on/4 s off; Sham: as above but with restricted transmission of magnetic pulse Assessment: ICIQ-UI-SF, 1 h pad test, PFM examination by perineometer, PGI-I, ICIQ-LUTSqol; follow-up at 1, 2, 5, 8, and 14 months	Long-term response was observed: The MS group showed significantly lower ICIQ-UI-SF values, lower frequency and severity of UI, and higher PGI-I values than in the Sham group. Objective and subjective cure values were higher in the MS group than in Sham group.
Gilling et al. (2009) [123]	Assessment of the effectiveness of extracorporeal electromagnetic stimulation in the treatment of SUI symptoms in patients undergoing unattended PFMT.	90 women with SUI, aged over 20: MS = 35 Sham = 35	For both: women were educated about PFM and received low-intensity PFMT program to home practice. MS: 3 times a week for 6 weeks 10 min at 10 Hz, 3 min rest, 10 min at 50 Hz Sham: same as above but with blocked transmission of magnetic waves through an aluminum plate Assessment: 3-day bladder diary; 24-h pad test, CVM, and perinometry for muscle score, I-QOL, KHQ, video-urodynomics	Both MS and Sham patients, performing only unattended PFMT, achieved improvement in the intensity of UI symptoms (pad test) and quality of life. There were no statistically significant differences between the MS and Sham groups, suggesting a similar efficiency of the MS stimulation parameters used in these studies for unattended PFMT.
Yamanishi et al. (2014) [113]	Evaluation of the effectiveness and safety of MS in the treatment of UUI in women.	Women with OAB: Active = 94 Sham = 49	Time: 25 min, twice a week, for 6 weeks Magnetic field parameters: Active: 560 mT, 300 μs, 10 Hz Sham: 114 mT, 300 μs; 1 Hz, 5 s on/5 s off cycle, Assessment: The bladder diary; IPSS QOL	MS effectively reduced UUI symptoms in patients with OAB. A significant reduction in the leak per week and urgency per 24 h and increase in mean and maximum voided volume per micturition was noted in the active MS group, but not in the Sham group.
Yamanishi et al. (2019) [112]	The effect of magnetic stimulation on the treatment of SUI in women refractory to PFMT.	For both: women with no effects of PFM training for more than 12 weeks Active =18 Sham =12	For both: 20 min, once per week for 10 weeks Magnetic field parameters: 5 s on/5 s off cycle, 300 μs Active: 272 mT, 50 Hz, Sham: 114 mT, 1 Hz Assessment: urodynamic examination, the voiding diary, the 24 h pad test, ICIQ-SF, ICIQ-QOL, ALPP	MS effectively reduced UI symptoms in PFMT-resistant women with SUI. A significant reduction in the number (voiding diary) and intensity (pad test) of UI episodes and increase in ALPP was noted in the active MS group, but not in the Sham group.

ExMI: extracorporeal magnetic innervation; SUI: stress urinary incontinence; QUID: The Questionnaire for Urinary Incontinence Diagnosis; GSES: The General Self-Efficacy Scale; RUIS: The Revised Urinary Incontinence Scale; BDI: Beck Depression Inventory; PFMT: pelvic floor muscle training; KHQ: King’s Health Questionnaire; MS: magnetic stimulation; SLPP: The stress leak-point pressure; CVM: Circumvaginal muscle rating score; I-QOL: Incontinence Quality-of-Life Scale; PGI-I: Patient Global Impression of Improvement; ICIQ-LUTSqol: ICI Questionnaire-Lower Urinary Tract Symptoms Quality of Life; IPSS QOL: International Prostate Symptom Score-Quality of Life; ICIQ-SF: International Consultation on Incontinence Questionnaire—Short Form; ICIQ-QOL: International Consultation on Incontinence Questionnaire—Quality of Life; ALPP: Abdominal leak-point pressure.

**Table 5:** Characteristic of selected studies on the effects of whole-body vibration (WBV) training on the pelvic floor muscle (PFM) activity and/or severity of urinary incontinence (UI) symptoms.

Reference	Main Objective	Patient Characteristic	Study Description	Outcome
Lauper et al. 2009 [134]	Verification of the effectiveness of sinusoidal and stochastic resonance WBV on PFM activation in healthy and postpartum women.	Control group: 23 healthy women; aged 18–40 Post-partum group: 26 women, aged 18–40; 8 weeks to 1 year after delivery with a week PFM (0–3 in Modified Oxford Scale)	Verification of PFM activity in sEMG during different type and intensity of vibration measured with and without MVC of PFM. S-WBV: 5, 10, 15, 20, or 25 Hz with 2 or 4 mm amplitude. SR-WBV: 2, 4, 6, 8, 10, or 12 Hz. Assessment: PFM activity in sEMG	Both vibration methods effectively activated the PFM, minimal parameter is 6 Hz for SR-WBV and 15 Hz/4 mm for S-WBV. SR-WBV induced significantly higher level of PFM activation than sinusoidal WBV, and exceeds the value of MVC in postpartum group (127%; 12 Hz).
Luginbuehl et al. 2012 [130]	Comparison of continuous and intermittent stochastic resonance WBV (SR-WBV) to determine the optimal vibration methodology for PFM activation.	Group 1: 28 women (8 weeks to 1 year after delivery) aged 18–45; Group 2: 22 women (more than year after childbirth or nulliparous) aged 18–80; All with SUI	SR-WBV = 8 Hz Continuous: 3 sets of 60 s WBV with 60 s rest between sets; Intermittent: 3 sets of 12 × 5 s WBV with 10 s rest between WBV and 60 s rest between sets. Assessment: PFM strength in sEMG and Modified Oxford Scale	The training provoked PFM activation at only 50–70% of MVC. There were no differences in PFM activation between the tested modalities (continuous versus intermittent). For practical reasons, the authors suggest the use of continuous vibration in clinical practice.
Farzinmehr et al. 2015 [135]	Assessment of the effectiveness of WBV training in improving the strength of the PFMs in women with SUI.	RCT: 43 women with SUIWBVT group—21; PFMT group—22;	Training for both: 3 times a week for 4 weeks (a diversified, progressive program). WBVT: 30–50 Hz; 2.5–5 mm; rest 30–60 s PFMT: supervised training consisting of 3–4 sets of 15–20 repetitions with 60 s break between sets. Assessment: PFM strength in sEMG and Modified Oxford Scale, incontinence severity in VAS; Quality of Life—I-QOL	In both groups, a significant (*p* = 0.0001) post-training improvement was obtained in terms of reducing UI severity, increasing PFM strength, and improving the quality of life. In addition, there were no differences in the above parameters between the WBVT and PMFT groups, which proves the equal effectiveness of both methods.
Stania et al. 2015 [133]	Assessment of bioelectric PFM activation during low and high-intensity synchronous WBV in healthy women.	RCT: 33 continent nulliparous LI-WBV group: 12, aged 22.4 ± 1.6; HI-WBV group: 10, aged 21.4 ± 1.7; CTR w/o WBV: 11, aged 22.7 ± 1	Three sets of static WBV exercises (30, 60, or 90 s), performed in a random order, with a 60 s rest between them: LI-WBV group: 20 Hz, 2 mm HI-WBV group: 40 Hz, 4 mm. Assessment: PFM sEMG	Long-term (60, 90 s), high-intensity (40 Hz) WBV training significantly caused a significant increase in PFM activation, which was not observed in low-intensity training.

SR-WBV—stochastic resonance whole-body vibration; PFM—pelvic floor muscle; RCT—Randomized Clinical Trial; LI-WBV—low-intensity whole-body vibration; HI-WBV—high-intensity whole-body vibration; WBVT—whole-body vibration training; PFMT—pelvic floor muscle training; I-QOL—The Incontinence Quality-of-Life Questionnaire; VAS—Visual Analog Scale.

**Table 6:** Characteristic of selected studies on the effects of direct vibration on the pelvic floor muscle (PFM) activity and/or severity of urinary incontinence (UI) symptoms.

Reference	Main Objective	Patient Characteristic	Study Description	Outcome
Ong et al. 2015 [137]	Effectiveness of PFM exercises combined with the Vibrance Kegel Device (VKD) compared to PFMT alone in women with SUI	Two groups: PFMT only: 19; PFMT + VKD: 21For both: women with SUI, age over 18 years	For both: 16 weeks, control and PFM re-education at 0–4–16 week. PFMT group: daily PFM exercise according to the protocol and 1 per month with a therapist (20 min); PFMT + VKD group: daily PFM training with VKD as BF. Assessment: Australian Pelvic Floor Questionnaire (APVQ), Modified Oxford Scale for PFM activity	In the PFMT + VKD group, SUI score (APVQ) was improved after just 4 weeks. The values equalized with those of the PFMT group at week 16. In addition, after 4 weeks, the strength of PFM in the PFMT + VKD group was significantly higher than that in the PFMT group, which was also maintained at week 16. VKD can be a valuable and effective tool to support PFMT.
de la Torre et al. 2017 [138]	Assessment of the effectiveness of multimodal vaginal toning therapy in improving of bladder symptoms and quality of life in women with postpartum SUI	48 women with postpartum SUI, aged 30–59	Stimulation: daily for 45 days, at home for up to 10 min of intravaginal multimodal stimulation (light therapy: 662–855 nm; heat: 41 °C; vibration: 80–110 Hz). Assessment before and post-treatment: UDI-6, IIQ-7, FSFI, FSDS-R, Oxford Scale for PFM strength, 1 h pad test	An improvement of over 50% was noted after therapy in the following areas: loss of urine in 1-h pad test in 84% respondents; improving the quality of life (UDI-6) in 92% of respondents and in 85% respondents (IIQ-7). The strength of PFM also increased significantly. The level of patient satisfaction with therapy reached 83%.
Rodrigues et al. 2019 [139]	Comparison of the effect of intravaginal vibration stimulation (IVVS) with intravaginal electric stimulation (IVES) in women with PFM dysfunction (without voluntary PFM contraction)	Group IVVS = 21 Group IVES = 21 Women’s age> 18 years and Grade on the Modified Oxford Scale = 0 or 1	For both: once weekly (20 min) for 6 weeks IVVS parameters: 95 Hz; amplitude: 1.5 mm; work/rest time: 8/16 s IVES parameters: 50 Hz; pulse width: 300 ms; work/rest time: 8/16 s; the current was adjusted to the patient’s individual tolerance. Assessment of PFM: Modified Oxford Scale, New PERFECT scale; ICIQ-SF	After 6 weeks, both groups showed improvement in UI as measured by ICIQ-SF, without intergroup differences. There were no post-therapeutic differences between IVVS and IVES in terms of PFM functions such as endurance, fast contraction, and repetition. However, after vibration training, the PFM strength significantly increased relative to that in the IVES group.

PFM—pelvic floor muscle; VKD—Vibrance Kegel Device; SUI—stress urinary incontinence; PFMT—pelvic floor muscle training; APVQ—Australian Pelvic Floor Questionnaire; UDI-6—Urogenital Distress Inventory Short Form; IIQ-7—Incontinence Impact Questionnaire—Short Form; FSFI—Female Sexual Function Index; FSDS-R—Female Sexual Distress Scale—revised 2005; IVVS—intravaginal vibration stimulation; IVES—intravaginal electric stimulation.

**Table 7:** Quality evidence of physiotherapy techniques in UI decisions in selected reviews.

Technique	Level of Evidence	GRADE	Authors
PFMT	I	A	Dumoulin et al. 2018 [141]
Electrical Stimulation	IIa	A	Stewart et al. 2017 [142]
Biofeedback	IIb	A	Bø 2012 [143]
Magnetic Stimulation	IIb	B	He et al. 2019 [104]
Body Vibration	IIb	B	Guedes-Aguiar et al. 2019 [129]
